# The importance of values in evidence-based medicine

**DOI:** 10.1186/s12910-015-0063-3

**Published:** 2015-10-12

**Authors:** Michael P. Kelly, Iona Heath, Jeremy Howick, Trisha Greenhalgh

**Affiliations:** Institute of Public Health, University of Cambridge, Forvie Site, Robinson Way, Cambridge, CB2 0SR UK; Royal College of General Practitioners, London, UK; Nuffield Department of Primary Care Health Sciences, University of Oxford, Oxford, UK

**Keywords:** Evidence-based medicine, Values, Medical ethics

## Abstract

**Background:**

Evidence-based medicine (EBM) has always required integration of patient values with ‘best’ clinical evidence. It is widely recognized that scientific practices and discoveries, including those of EBM, are value-laden. But to date, the science of EBM has focused primarily on methods for reducing bias in the evidence, while the role of values in the different aspects of the EBM process has been almost completely ignored.

**Discussion:**

In this paper, we address this gap by demonstrating how a consideration of values can enhance every aspect of EBM, including: prioritizing which tests and treatments to investigate, selecting research designs and methods, assessing effectiveness and efficiency, supporting patient choice and taking account of the limited time and resources available to busy clinicians. Since values are integral to the practice of EBM, it follows that the highest standards of EBM require values to be made explicit, systematically explored, and integrated into decision making.

**Summary:**

Through ‘values based’ approaches, EBM’s connection to the humanitarian principles upon which it was founded will be strengthened.

## Background

The first decades of Evidence Based Medicine (EBM) were devoted to developing the science of clinical epidemiology and improving the technical means of applying its principles and tools consistently and efficiently. The underpinning research base has been built by refining systematic, rule-bound approaches such as health technology assessment (HTA) [1], comparative effectiveness studies [2], systematic literature review [3], and by ensuring rigorous standards for reporting such studies [4].

These approaches are academically defensible and reproducible but can also be painfully arcane in their procedural and technical detail. Many clinicians have appreciated the increasingly impressive armoury of tools developed to support the practice of EBM but have felt simultaneously overwhelmed by their imperatives. The strong (and perhaps necessary) focus on technical procedure – how to do ‘robust’ research, how to synthesise data from primary studies, how to apply the findings in practice – has created the impression that EBM and its underpinning methodologies are concerned exclusively with matters of fact in an objective scientific environment, with confounders and bias either eliminated or carefully controlled for.

In this paper, we develop an argument which builds on the work of some of the early critics of EBM [5–8] and draws on philosophical and sociological writings [9, 10] to suggest that the methodological rules of EBM, and the research that underpins them, are laden with largely unacknowledged values. In doing so we explore in detail the meaning of the term values. The protagonists of EBM have long recognised that *patient values* and circumstances must be taken into account when making clinical decisions [11]. Yet for years, this aspect of EBM was given relatively little systematic attention in the movement’s main writings, textbooks, and courses. More recently some authors have shown how patient values and evidence go hand in hand in the decision-making process [12–16].

However the importance of values extends beyond the point-of-care decision with an individual patient. Values infuse evidence (in all sciences) at many levels. EBM, like all science, is *necessarily* value-laden. Not only is eliminating values from the scientific method – in general and the EBM process in particular – impossible, but in trying to do so, researchers may introduce new (mostly covert and unacknowledged) biases. By embracing and acknowledging values and exploring them seriously, we anticipate that EBM will achieve a more mature, and socially useful, status.

Interestingly, values were at the very heart of EBM when the movement began. Cochrane’s original argument was all about doing medicine that was in patients’ interests [17]. Sackett et al’s widely-cited description of EBM places particular emphasis on the best interests of the patient [18]. Using unbiased evidence was by definition beneficent because medical interventions are risky and using the best evidence offers patients protection both from medical incompetence and the (at times) overblown claims of Big Pharma [19]. Treatments about which there is medical and scientific uncertainty were framed as potentially harmful (‘maleficence’). EBM has positioned itself as the guarantor of not doing harm; to do anything other than EBM is tantamount to maleficence. Ironically, much of the recent backlash against EBM has been on the grounds that things have gone too far and that the slavish following of ‘evidence based’ guidelines poses threats to patients [20, 21]. We return to this issue below, but draw readers’ attention to the fact that EBM arose out of very real value and ethical concerns and values remain intrinsic to the enterprise.

## Discussion

### What are values?

Science aspires to be about the world *as it is*; values are about the world *as it ought to be*. Science seeks to get as close to the reality of the world as possible. Yet no matter how sophisticated our measurements become, we remain limited in our ability to access the truth because of our fallibility as observers and because of the intrinsic technical limitations of the instruments we use to do the observation. True essences if they may be said to exist at all are the province of philosophy, metaphysics and theology. What scientists are able to observe should not be confused with truth.

Contrary to popular assumption, then, neither essences nor truth are the territory of science and EBM. The world of empirical science is the world as it appears to be, revealed through our (imperfect) observational apparatus and methods. As practising scientists or doctors we must be humble about what we know, acknowledging our (and our tools’) fallibility. Opening our empirical work to refutation (by repeating experiments ourselves, and by inviting others to replicate our work) is the basis of good scientific practice.

The world as we think it *ought to be* is the world of values. Different people will have different values, and it is very hard to resolve value-based disagreements on the basis of scientific evidence. But values are ever present. Our hopes, beliefs, politics and religions, about which we (appropriately) feel emotions, provide us with the frame or the lens with which we see the world, our ambitions for the future and our understanding of the past. Despite the caricature of the passionless objective (often male) scientist in a white coat, the questions scientists decide to ask, the methods they select, and the way they interpret results are chosen through a filter of often unacknowledged and subconscious values [22].

Values can be thought of as a form of psychological heuristic [23]. Psychologists argue that our thinking is governed by two systems, the automatic and the reflective [24]. Our automatic responses are to cues in our environments, whereas our reflective system processes information, assesses costs and benefits and reasons before we take action. Cues take many forms but one type of cue consists of ready made quick answers to complex problems [23]. Our values can be seen as acting as short cuts and providing immediate answers to issues and problems we choose not to explore in detail. Scientists and doctors are no less prone to using heuristics than anybody else. Their training emphasises the rational and reflective side of their minds and in much day-to-day practice the reflective is dominant, but unacknowledged values will tend to act as heuristic devices. It is vitally important to avoid the trap of easy heuristic thinking. This does not deny the importance of values, but requires us to be wise to the ones we hold.

Such necessary wisdom is bolstered by another framing of values: not as the reflective ‘side’ of our psychology (in contrast to the rational ‘side’) but as the essential *texturing* of everything we perceive, believe and aim for. Nussbaum (among others) has emphasised that all observed facts are value-laden; how we feel about an issue emotionally, which is derived explicitly or implicitly and more or less consciously from our values, transforms a flat cognitive landscape into a three-dimensional terrain through what she calls ‘geological upheavals of thought’ [25]. Hence, values do not make our decisions and actions less rational. On the contrary, they give them meaning, significance and moral worth.

Different values underpin different priorities and different kinds of ethical judgements. Consider the example of a child whose birthday is approaching. The child’s father (driven by economic values) calculates that the child is likely to receive the best value in presents if he invites the 10 richest children in the class to his party. The child’s mother (driven by egalitarian values) considers that her son should invite the 10 children who have been invited to the fewest parties so far this year, since this would spread out the joy of party-going more fairly. The child (driven by deontological values) says *he* wants to invite his five closest friends because he feels a stronger duty to these individuals than to other classmates whom he hardly knows. None of these competing perspectives on whom to invite to the party is *incorrect*, but each is driven by a different set of values and makes sense in relation to that set of values.

It is helpful to distinguish between values and ideologies. An ideology is broader than a value and comprises a scheme of ideas relating to the conduct of a group or society. When ideologies become entrenched, people’s beliefs and values (which are often varied and changing) are translated into fixed systems of thought, unamenable to scientific tests or arguments that might challenge them. The process by which (individual) values are shaped into (group) ideologies is inevitably political and shaped by the powerful, whose intention is to control others [26].

Like values, ideologies find expression in language. At the level of the individual, language is open-ended and flexible, changed by each usage and infinitely adaptable to human aspiration. In a healthy democratic society, shared values are arrived at by deliberation and argument – and these shared values are an important source of social cohesion and stability. But when (and to the extent that) group values become ideologies, deliberation is replaced by iteration and, eventually, convergence in the interests of the powerful. Within a dominant ideology, the values of minorities are often overlooked, and may not even be recognised or measured.

The EBM and guidelines movements have sometimes been accused of ideological behaviour – that is of imposing a narrow, rule-based and overly technical approach to clinical practice and research; of seeking to control language; of suppressing dissent; and of dismissing alternative framings of problems and solutions [27, 28]. Some exponents of EBM remain of the view that values can and should be controlled for as removable sources of bias. In a paper on how politically controversial electronic patient record systems should be evaluated, for example, the two authors argued that *“health information systems should be evaluated with the same rigor as a new drug or treatment program, otherwise decisions about future deployments of ICT [information and communications technology] in the health sector may be determined by social, economic, and/or political circumstances, rather than by robust scientific evidence”* [29]. They argue for strictly controlled studies in which scientific findings can be generated in an environment untainted by societal, cultural or political perspectives. An alternative view is that scientific findings are meaningless unless these societal, cultural and political perspectives are taken into account – since such values-based influences necessarily frame the problem and shape the research questions and the interpretation of findings [30].

We believe that EBM has made significant progress in recent years (although evidence that EBM itself has had any positive benefit is notoriously lacking [31]). Many of its leading protagonists now argue for a more interdisciplinary stance that accommodates values and seeks to combine these with best evidence to achieve such goals as compassionate, patient-centred care and science for the public good [15]. As the core ideas of EBM have expanded into areas like public health and social care [32, 33], it has become a much broader and pluralistic endeavour than it was when it all began. Presently EBM is engaged in a process of critical self appraisal [21] and addressing the question of values head on is one of the tasks to be undertaken as it develops further.

### The value questions in evidence based medicine

Values have a critical influence in all aspects of EBM. Below we consider some key elements in turn.

1. The role of values in deciding which questions to ask
  Values strongly influence decisions about which technologies to develop in Phase I basic science research (which in turn influences which ones subsequently appear as evidence based therapeutic options).
  Of course, drugs and other technologies that eventually get to be appraised have come through numerous hurdles and only a tiny minority get as far as being tested in clinical trials; but decisions about which treatments to assess are necessarily political and economic and therefore value-based as well as scientifically-based. Much of the agenda is set by the life sciences and pharmaceutical industries because they determine which products are brought to market and which products to seek authorisations for which indications [19, 31, 34].
  While a case can be made that this system supports innovation in industry, such innovation may map poorly to need in terms of the burden of disease nationally or internationally – and hence represent an opportunity cost for patients and the health service. If medical science is defined purely in terms of commercially viable innovations tested in randomised trials, there will be less funding available to be spent (and less political will to spend it) on preventive non-drug interventions and public health measures such as food labelling or the walkability of the built environment.
  These choices are the consequences of values held by key actors in the system – particularly the perspective that health is best delivered through measures that also generate innovation and wealth [35]. Such an approach to the funding of scientific research may be a far cry from the disinterested pursuit of the greater societal good [36, 37]. An alternative perspective places greater value on equity, justice and fairness – and emphasises the potentially negative consequences of innovation and profit [31, 37]. In the UK, the HTA programme was specifically set up to counterbalance the commercial biases of the relevant industries and many HTA assessments are done on orphan drugs, diseases or procedures, or ones that offer no profit to industry [1]
  It is not our purpose in this paper to support a particular position on this question, simply to emphasise that both sides are expressing values, not making logical deductions from neutral evidence. Only when the value-ladenness of resource allocation for scientific research is acknowledged can democratic societies begin to deliberate on which values *should* drive this allocation.
2. The role of values in selecting methods for identifying and appraising research evidence
  Over the last three decades, EBM has honed a series of methods to measure and appraise the technologies it evaluates. This has been an area of considerable scientific and methodological advance. The importance of the clinical trial as the method of choice to determine efficacy is now well established, and the analytic techniques to conduct trials robustly are now so sophisticated that expertise is clustered in clinical trials units [38].
  However, the choice of methods and the topics to which they are applied are not value neutral decisions. They have epistemic consequences because they not only determine the nature and type of knowledge which is generated by these methods, but also in a very important sense they define what is and isn’t admissible or counted as knowledge and evidence in the first place. So despite the considerable efforts that have gone into methodological refinements of the RCT for example, trials may still reveal a skewed version of biomedical science. Their design means they are a controlled experiment, oriented to generating an average result in an unconfounded population sample removed from the messiness of real life. The technical method is pristine, but the degree to which the findings might apply to the atypical patient (e.g. with multi-morbidity or complex circumstances) and/or the atypical service setting (e.g. the hospital without a rapid-access chest pain clinic or the general practice whose ECG machine is broken) requires judgements that the science does not supply [8]. The epistemic consequence of taking a population or sub-population approach and looking at average results in these populations opens up a gap between the empirical evidence generated in the trials and the needs of individual patients [5–7]. Although the rhetoric of EBM argues for the importance of the individual patient, the failure to note that the choice of methods has profound consequences for the generation of data means that the significance of this gap is systematically overlooked [34].
  At issue here is not merely the trade-off between methodological purity and real-world applications, but also the extent to which internally valid randomized trials are privileged over pragmatic trials, and the extent to which the randomised trial in general is privileged over other research designs in which the clinical judgement of the practitioner is factored in rather than controlled out [39, 40]. The tension between objective experimental purity and subjective, case-based clinical judgement is often acknowledged by writers within and critics of the EBM movement [5–8, 11, 13], but there is a significant (and as-yet largely unaddressed) research agenda to unpack and explore this tension systematically at both a philosophical and an empirical level.
3. The importance of patient values in clinical decision-making
  Evidence-based medicine is committed to the integration of patient values and circumstances with evidence and clinical expertise [18]. Yet EBM advances have mostly aimed at methods for reducing bias in clinical trials and systematic reviews, with very little effort being spent investigating methods to elicit patient values, and how to integrate them into clinical decisions [12, 41].
  There is another way in which values play a role in treatment decisions. Even if a treatment meets the cost utility threshold (that is, is shown to be both effective and cost-effective compared to placebo), another treatment – or indeed no treatment at all – may still be preferable. Decisions about which treatments to offer from among a range of available alternatives are value laden. These values are most apparent when the treatment modalities differ greatly, for example surgical versus non-surgical interventions. One patient may prefer bariatric surgery to treat obesity, while another might opt for exercise. A cancer patient may choose the comfort of palliative care even if it means giving up on the chance of living longer that might be provided by aggressive chemotherapy. A patient with an active sex life might not want to take a drug that brings the risk of sexual dysfunction, and instead opt for another drug, even if it is likely to be less effective. While evidence plays a role in these choices, evidence alone is insufficient: patient values play an equally important role.
  The vast majority of evidence-based guidelines are derived from research into a single disease state. However, many people and most of those aged over 75 have more than one condition. Multiple guidelines may be applied, suggesting multiple medications and other interventions. The net result can be a degree of polypharmacy which becomes both burdensome, potentially dangerous [42], and non evidence-based – since patients with multi-morbidity are almost invariably excluded from clinical trials [43] Patients may also make an informed choice to reject medication that needs careful monitoring because of the necessity of frequent clinic visits and/or blood tests. Most preventive interventions carry only a small chance of benefit for the individual and patients may decide that this is insufficient to justify the taking of regular medication indefinitely – especially if they have previously experienced significant side effects from medication. Careful attention to the hopes, aspirations and values of each individual patient will result in very different treatment decisions.
4. The importance of clinician values in prioritising (so-called) evidence-based tasks
  The concerns of patients in the clinical setting are the focus of work by Values-Based Medicine (VBM) researchers, who explain how to integrate patient and practitioner values into clinical decision-making [12, 44]. But to date, VBM researchers have tended to restrict their focus to the specific decision-point in the clinical consultation and do not discuss the many ways in which considering values is important at all the other stages of EBM.
  Whilst patients’ values are paramount, clinicians’ interpretation and application of evidence are strongly influenced by their own values. Doctors and nurses are professionals – that is, they operate in a closed shop with high entry standards, an advanced knowledge base and a strong sanction from society to act ethically and in the best interests of their patients [45]. Clinicians who resist evidence based recommendations may do so because they believe those recommendations conflict with over-riding principles such as the need to protect confidentiality, advocate for the vulnerable, or avoid causing harm [46].
  In addition, doctors need to balance the time available for an individual patient with the time left to spend on other patients. One evidence-based recommendation that generates large amounts of work for clinicians inevitably threatens other evidence-based interventions since there is only a finite number of hours in the day. The recent NICE decision to lower the threshold for statin prescribing, for example [47], raises issues at the system level. The decision implies that careful shared decisions will need to be made with thousands more asymptomatic people and the recommendation in the guideline seems to take little account of, and appears to attach no value to, the amount of time and effort this involves for already hard-pressed clinicians. How should we assess the value attached to such opportunity costs?
5. Values in the broader sense – is EBM delivering on its promise?
  From the outset, EBM has been dominated by questions of efficiency and value for money [48, 49]. Indeed, a key achievement of EBM in England’s publicly funded health system is that both clinical efficacy and cost effectiveness must now be demonstrated before new treatments are funded. But preferences for efficiency and value for money are *value* preferences, not scientifically neutral and dispassionately observed matters of fact [34, 50]. In the methods used to determine value for money, the value (stemming from utilitarian philosophy) *is* efficiency [49]. From the moment Archie Cochrane linked questions of clinical effectiveness to cost effectiveness [17] and cost utility analysis was chosen as the basis for assessing value for money, EBM and HTA have been framed within the utilitarian philosophical tradition.
  Utilitarianism is premised on the view that actions are good insofar as they maximize benefit for the greatest number [51]. This is not necessarily congruent with what is in the best interest of an individual patient [34]. Because the utilitarian philosophy of resource allocation is so pervasive in our society, it is not necessarily obvious that this core assumption *is* a value. When utilitarian philosophy is applied to health we assume that we want the greatest health gain for the greatest number of people. An intervention is deemed to beneficent if it achieves this but to do so we have to make a value judgment about how much that intervention is worth. However, there are different ways of assessing how much an intervention is worth.
  For example, the time I spend with my elderly mother is supposedly efficient because I know she is going to die soon, so I place high value on the time I have left with her. If I did not value that time, I might describe the same encounter as an inefficient use of my time. On the other hand if I use standard cost utility analysis, I am using a mechanism that determines value for money for society as a whole (the greatest happiness for the greatest number), not for individuals. Inevitably, some individuals lose out because no matter how much money is spent there will always be demand that is unmet and treatments that cannot be funded – resources are finite. So although cost utility analysis provides an open and transparent method for resource allocation, it is based on a set of values.
  Utilitarians believe that the greatest health gain for the greatest number is efficient and that whilst some will not benefit as a consequence of the methods used to determine allocation, this is a price worth paying for the greater good (and morally the best approach because it is the most efficient) [34]. A competing view proposes that equity should be paramount and that it is unjust and unfair that there are winners and losers as a consequence of whatever method of resource allocation is used. The drive to improve efficiency (to achieve the greatest health benefit for the greatest number) leads to inequities since not all treatments can be provided for all people and an *a priori* judgement has been made about which treatments will be funded on the basis of the principles of efficiency of national resource allocation. Efficiency can be unfair because not everyone individually will get what is right or appropriate for him or her. The cost utility approach tends to have little or no impact on patterns of need in the population or on health inequalities and at the very least reinforces the *status quo* [52].
  However, equity itself can mean a number of different things depending on one’s values: it can mean everybody is treated in exactly the same way regardless of who they are or what is wrong with them but it might also mean that those in greatest need should have the first call on resources. Anderson draws our attention to the fact that the concept of equality depends on the underlying political values [53]. People have different values in relation to the extent to which they think society should be flatly egalitarian, or whether societal assets should be distributed unequally according to need. This is difficult because the needs of different age groups and social classes are very varied and measuring need is practically very difficult [52]. These questions are not resolvable with reference to science. Our views about equity (and efficiency) are value preferences, which will influence our methodological preferences too, and our interpretation of the findings that our scientific methods reveal.
  Despite all this, within the immediate realities of clinical practice, deontology predominates and tends to marginalise both utilitarian and egalitarian values. The task of the clinician is to engage with the needs and values of each individual patient. Within any consultation, the moral obligation of the professional is to do his or her best for that particular patient and so the values of clinicians inevitably become primarily deontological [45, 54–56].
  This value-based commitment of the clinician to the individual patient is poorly understood and little appreciated by those policymakers whose priorities are situated at the population or societal level, and vice versa. Yet arguably, if front-line clinical practice were not strongly rooted in deontology, patients could find themselves unable to trust clinicians. This is because patients would be likely to worry that practitioners whose practice was *not* rooted in deontology would legitimately be concerned that their clinicians might set aside individual patient values and interests in the name of greater public good. The ensuing lack of trust would be less efficiency at a societal level. Recent empirical research on resource allocation decisions in the UK National Health Service, for example, has illustrated the tensions that can arise when a clinician, driven by a deontological commitment to his or her patient, appeals against a local policy not to fund a treatment that has a high cost per quality-adjusted life year [57, 58]. Many UK providers have set up panels to consider such appeals, known as ‘individual treatment funding requests’. Panels’ decisions were strongly utilitarian, since they were responsible for allocating a set budget to provide healthcare for a geographically defined population. But clinicians in these studies invariably dismissed or downplayed arguments about fairness or equity and constructed their appeals in terms of their professional commitment to do the very best for the particular individual whose case they were presenting. Unsurprisingly, the studies revealed no simple formula for resolving these disputes.

## Conclusions

EBM has generated substantial advances in methodology that have allowed us to distinguish between helpful and harmful treatments, identify the major problems with publication bias, and surface and address industry conflicts of interest. Unfortunately, the predominance of technical progress has also served to support the myth that EBM is value neutral. The focus on technical methodologies has obscured the equally important issue of values and, in turn, the way values impinge on judgements and the processes of interpretation of all steps in the EBM process. While technical progress must continue, at least some effort in the next decades should be given to exploring questions of value. This is not just a philosophical or methodological point; it is of profound practical importance.

Values may act as heuristics – shortcuts in our thinking of which we are barely aware – which get us to quick answers to complicated problems. They form the lens through which we perceive and act on our world. Values are often tricky to pin down because they are such a pervasive part of things we take for granted. A necessary first step towards achieving this is to make our values as explicit as we can, so that we can reflect on them individually and deliberate on them collectively [42]. Until EBM reconnects with its values and allows that its purpose is to extend human capabilities within a constrained environment, it will remain open to the accusation that it has lost its soul and come adrift from its founding humanitarian principles.
